# Overview on the Design of Magnetically Assisted Electrochemical Biosensors

**DOI:** 10.3390/bios12110954

**Published:** 2022-11-01

**Authors:** Yong Chang, Yanyan Wang, Jingyi Zhang, Yuejiao Xing, Gang Li, Dehua Deng, Lin Liu

**Affiliations:** College of Chemistry and Chemical Engineering, Anyang Normal University, Anyang 455000, China

**Keywords:** electrochemical biosensor, magnetic particle, immunosensor, DNA biosensor, aptasensor, homogeneous assay

## Abstract

Electrochemical biosensors generally require the immobilization of recognition elements or capture probes on the electrode surface. This may limit their practical applications due to the complex operation procedure and low repeatability and stability. Magnetically assisted biosensors show remarkable advantages in separation and pre-concentration of targets from complex biological samples. More importantly, magnetically assisted sensing systems show high throughput since the magnetic materials can be produced and preserved on a large scale. In this work, we summarized the design of electrochemical biosensors involving magnetic materials as the platforms for recognition reaction and target conversion. The recognition reactions usually include antigen–antibody, DNA hybridization, and aptamer–target interactions. By conjugating an electroactive probe to biomolecules attached to magnetic materials, the complexes can be accumulated near to an electrode surface with the aid of external magnet field, producing an easily measurable redox current. The redox current can be further enhanced by enzymes, nanomaterials, DNA assemblies, and thermal-cycle or isothermal amplification. In magnetically assisted assays, the magnetic substrates are removed by a magnet after the target conversion, and the signal can be monitored through stimuli–response release of signal reporters, enzymatic production of electroactive species, or target-induced generation of messenger DNA.

## 1. Introduction

Analytical devices are of great importance for early clinical diagnosis, which requires fast and cost-effective analysis of biological samples. To date, different sensing techniques have been developed, including mechanical, optical, and electrochemical biosensors. In the past decades, electrochemical biosensors have received great attention in light of their advantages of simplicity, portability, and high selectivity, as well as sensitivity [1]. However, the use of a traditional solid electrode for the immobilization of recognition elements (e.g., antibodies, enzymes, DNA, and aptamers) brings about several problems, thus hindering the practical applications of electrochemical biosensors [2,3,4,5]. For example, (i) biological macromolecules tethered on an electrode may shield the sensing surface, subsequently hindering the electron transfer and reducing the electrochemical signals; (ii) immobilization of biomolecules onto the electrode surface is time consuming; and (iii) the permanent immobilization of recognition elements on the electrode may limit the reproducible regeneration of biosensors. To address these problems, magnetic beads (MBs) and nanoparticles (MNPs) were introduced into the design of electrochemical biosensors as the solid phases for the recognition reaction [6]. The electrode surface can be easily renewed by adjusting the position of external magnet or polishing the electrode slightly. Moreover, it is effective to integrate MB-based electrochemical biosensors into arrays for automatic and high-throughput analysis [7].

With the fast development of nanotechnology, high-quality magnetic materials with different compounds and phases have been successfully prepared using various methods, such as co-precipitation, electron beam lithography, microwave-assisted synthesis, microemulsion, hydrothermal route, sonochemical synthesis, and biological route [8,9]. To reduce toxicity and improve stability of magnetic materials in vivo or in vitro, different surface coatings have been prepared on particle surfaces, including surfactants, proteins, polymers, siloxane, phospholipid micelles, inorganic metal nanomaterials, metal oxides, carbon nanomaterials, and metal organic frameworks [10,11]. To increase the half-life by protecting MBs/MNPs against degradation, the coatings can facilitate the conjugation of specific components, such as small molecules, biomolecules, and other nanostructured materials via electrostatic adsorption, covalent attachment, and affinity-based immobilization. The easy functionalization, excellent biocompatibility, and controlled movement by the means of external magnetic field endow MBs/MNPs with great potential in the field of bioassays. When being applied in biosensing, the modified MBs and MNPs can specifically and efficiently capture and separate various targets ranging from metal ions to cells with the aid of an external magnetic field, which is then re-suspended in a purified solution by removing the magnet [12,13]. In the electrochemical bioassays, MBs/MNPs mainly play four roles in biological applications on the basis of their unique characteristics. (i) They can be employed as electrode modifiers for the immobilization of biological macromolecules to improve the performances of electrochemical biosensors due to their excellent conductivity, large surface area, good biocompatibility, and abundant functional groups. The general immobilization methods include physical adsorption and magnetic assistance [14,15]. For instance, (aminopropyl)-triethoxysilane-coated MNPs, hybrids of multi-walled carbon nanotubes with MNPs, and MNP–room temperature ionic liquid composites have been deposited to immobilize DNA or enzymes on electrodes [16,17,18]. (ii) MBs modified with electroactive species (thionine or Thi and ferrocene or Fc), enzymes (horseradish peroxidase or HRP and glucose oxidase or GOx), and catalytic nanoparticles (PtNPs, AuNPs, bimetallic Cu@Au NPs, and Au@Pt NPs) can serve as the signal tags to enhance the detection sensitivity of electrochemical biosensors [19,20,21,22,23,24]. In addition, it has been suggested that MNPs can be used as the sacrificial probes to develop electrochemical biosensors by converting MNPs into Prussian blue through the reaction of Fe^3+^ and co-existing K_4_[Fe(CN)_6_] [25,26,27,28,29]. (iii) MBs can be used as the recognition reaction platform, on which the assay kinetics are fast due to the homogeneous dispersion state of MBs in the liquid phase. The final target-MB complexes are brought close to the electrode surface by magnetically assisted pre-concentration, followed by electrochemical detection. This can simplify the pre-treatment and detection procedures without centrifugation, filtration, and solid-phase extraction. (iv) MBs can be utilized to develop homogeneous biosensors, in which the targets are interacted with the recognition units on the surface of MBs in the liquid phase. Such interactions can trigger the release of certain mediate species (e.g., electroactive molecules, enzymatic products, and messenger DNA) that can be further determined by various electrochemical methods.

**Scheme 1:**
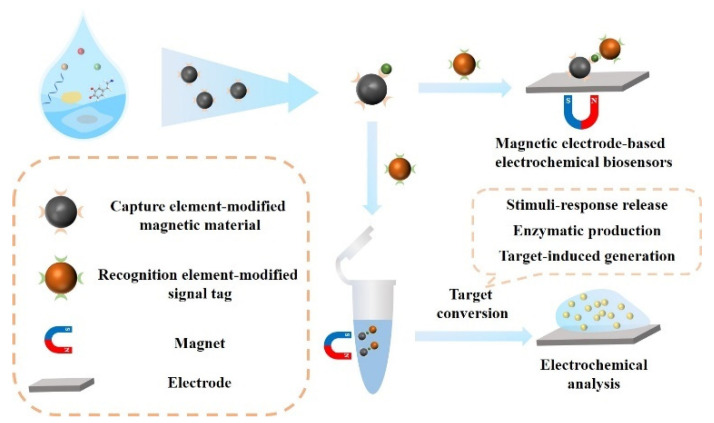
Schematic illustration of the strategies for magnetically assisted electrochemical biosensors involving magnetic materials as the platforms for recognition reaction and target conversion.

There have hitherto been several reports on magnetic biosensors [30,31,32,33,34,35]. For example, the progress in magnetic sensors for marine toxin detection and pharmaceutical and biomedical applications have been summarized by Tajik’s group and Ozkan’s group [36,37]. Hsing et al. reviewed the advancements and applications of micro- and nano-magnetic particles for biosensing [38]. Marfà et al. summarized the development of magnetic-molecularly imprinted polymers for electrochemical sensors and biosensors [39]. To the best of our knowledge, there is a lack of reports that systematically summarize the design of electrochemical biosensors involving magnetic materials as the platforms for recognition reaction and target conversion. In this review, we first summarize the advancements of immunosensors, DNA sensors, and aptasensors by collecting the magnetic conjugates with magnetic electrodes after recognition reactions (e.g., antigen–antibody, DNA hybridization, and aptamer–target interactions). Then, we discuss the design of electrochemical biosensors using magnetic materials as the target-conversion platforms (Scheme 1). After the target-conversion reactions, the magnetic substrates are removed by a magnet. The signal change can be monitored through stimuli–response release of signal reporters, enzymatic production of electroactive species, or target-induced generation of messenger DNA.

## 2. Magnetic-Electrode-Based Electrochemical Biosensors

In this section, we mainly focused on the achievements of magnetic-electrode-based electrochemical biosensors using MBs and MNPs as the recognition reaction platforms. The direct electrochemical detection of targets captured by MBs or MNPs on magnetic electrodes could be achieved on the basis of the redox activity or electrocatalytic ability of the targets themselves [40,41]. Moreover, the receptor–target complexes immobilized on the magnetic electrode solution interfacial region may hamper the electron transfer between redox mediator and electrode, inducing a change in the electrochemical impedance or capacitance [42,43]. These direct detection methods exhibit high simplicity, but they show low sensitivity because of the limited change induced by the relatively small size of antibodies and antigens. Thus, sandwich-type electrochemical biosensors with different signal amplification strategies have been explored to improve the sensitivity. They rely on the target-mediated formation or dissolution of sandwich complexes between the labeled detection elements and the acceptor-modified MBs or MNPs. Then, the magnetic conjugates were separated and transferred onto the electrode by an external magnetic field, thus producing a detectable electrochemical signal. On the basis of the type of acceptors, the magnetic-electrode-based electrochemical biosensors can be divided into three categories: immunosensors, DNA biosensors, and aptasensors.

### 2.1. Electrochemical Immunosensors

Electrochemical immunosensors are mainly performed on the basis of the signal changes induced by the immunoreactions between antibodies and antigens. On the basis of the reaction formats, they can be divided into competitive and sandwich-like immunosensors. Different signal labels have been proposed to transduce the recognition events into amplified electrochemical signals, including enzymes, nanomaterials, and DNA assemblies (Table 1).

#### 2.1.1. Enzymatic Amplification

**Figure 1:**
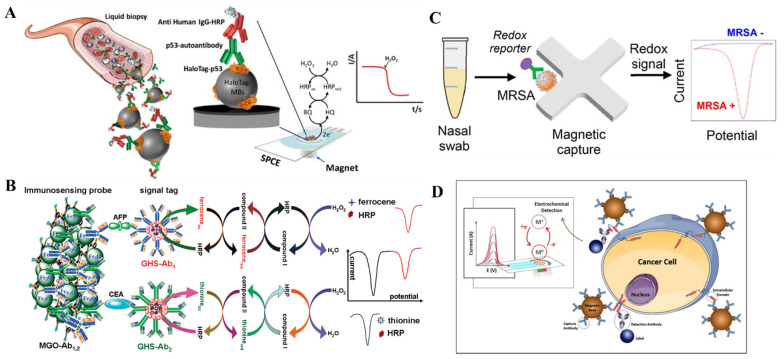
(**A**) Schematic illustration of the HaloTag fusion protein modified MB-based immunosensing platform for the amperometric detection of p53-specific autoantibodies. Reproduced with permission [57]. Copyright 2016, American Chemical Society. (**B**) Schematic illustration of a magneto-controlled HRP-based multiplexed immunoassay for the detection of carcinoembryonic antigen (CEA) and α-fetoprotein (AFP). Reproduced with permission [65]. Copyright 2011, American Chemical Society. (**C**) Schematic illustration of the bacterial capture and electrochemical detection based on antibody-modified MNPs and ALP. Reproduced with permission [67]. Copyright 2019, American Chemical Society. (**D**) Schematic illustration of the bacterial capture and electrochemical detection based on antibody-modified MNPs and ALP. Reproduced with permission [68]. Copyright 2020, Elsevier.

Enzymes have been extensively used in the fabrication of immunosensors because of their high catalysis efficiency and excellent specificity toward substrates [44,45]. After the capture of magnetic conjugates on the electrode, enzymes conjugated on the detection elements can accelerate the redox reactions and thus amplify the electrochemical signal [46,47,48]. For example, HRP acting as a signal tag in optical and electrochemical bioassays can catalyze the oxidation of substrates (e.g., hydroquinone or HQ and 3,3′,5,5′-tetramethylbenzidine or TMB) in the presence of H_2_O_2_ [49,50,51]. Pingarron’s group proposed many magnetic electrochemical immunosensors by using HRP-labeled antibodies as the signal labels [52,53,54,55,56,57]. As an example, shown in Figure 1A, they used a HaloTag fusion p53-protein-modified magnetic bead as the solid bioplatform for electrochemical assays of p53-specific autoantibodies [57]. Serum p53 autoantibodies were held by the HaloTag-p53-modified MBs and then recognized by HRP-conjugated anti-mouse IgG. After the immunocomplex-modified MBs were recruited on the surface of a disposable electrode with an external magnetic field, HRP catalyzed the redox reaction between H_2_O_2_ and HQ, thus generating an enhanced amperometric current. The biosensor exhibited higher sensitivity (440 times) than that of the conventional enzyme-linked immunosorbent assay. It could discriminate p53-seroreactive and nonseroreactive patients with low cost and short time. In addition, Castilho et al. reported a magneto immunoassay for the detection of *plasmodium falciparum* histidine-rich protein 2 related to malaria [58]. In this study, anti-HRP2-antibody-modified MNPs were used to capture HRP2 and HRP-labeled signal antibodies. After the capture of the resulting MNPs by a magneto electrode, HRP catalyzed the reaction between H_2_O_2_ and HQ, providing a strong electrochemical signal. The MNP-based electrochemical immunosensor exhibited good detection performance and low detection limit (0.36 ng/mL). Moreover, Perrotta et al. developed an electrochemical magneto-immunosensor for ochratoxin A determination using HPR-labeled ochratoxin A as the competitor [59]. After the competitive immunoreaction, the modified MBs were immobilized on the carbon-screen-printed electrode, and HRP catalyzed the oxidation of pyrocatechol by H_2_O_2_. This method was successfully used to detect ochratoxin A in red wine samples.

To improve the detection performance, an electrochemical signal can be further amplified by increasing the ratio of enzyme and signal antibody for each recognition reaction event. Typically, the polymeric HRP conjugates (poly-HRP) are used instead of a single HRP molecule to convert the bio-recognition event into a measurable signal [60,61]. Nanomaterials with a large surface area and good biocompatibility such as gold nanoparticles (AuNPs) and graphene oxide are usually utilized as the carries for loading of enzymes and antibodies [62]. Meanwhile, nanomaterials can enhance the electron transport between the electrode and the redox center of enzyme. For this consideration, Zhu et al. developed a magnetic electrochemical immunosensor for *Escherichia coli* O157:H7 detection by using neutral red-functionalized reduced graphene oxide to carry HRP and Au@Pt core/shell nanoparticles [63]. As a result, they were able to determine Escherichia coli O157:H7 with a linear range of 4.0 × 10^2^ to 4.0 × 10^8^ CFU/mL and a detection limit of 91 CFU/mL under the synergetic catalysis of Au@Pt NPs and HRP. To accelerate electron transfer and decrease reaction time, Tang et al. proposed an electrochemical immunosensor for the detection of carbohydrate antigen CA125 by employing magnetic silica beads to load HRP and Thi [64]. By using two different electroactive molecules (Thi and Fc) as the redox mediators, Tang et al. reported a flow-through magneto-controlled multiplexed immunosensor for the simultaneous detection of carcinoembryonic antigen and α-fetoprotein using nanogold hollow microspheres (GHS) to carry HRP and electroactive molecules [65]. As displayed in Figure 1B, the two capture antibodies of anti-carcinoembryonic antigen (Ab_1_) and anti-α-fetoprotein (Ab_2_) were co-immobilized on the Fe_3_O_4_-nanoparticle-decorated graphene nanosheets (MGO-Ab_1,2_). After the immunoreactions in the presence of targets, the catalytic reduction of H_2_O_2_ was accelerated by HRP with Thi and Fc as electron mediators. Then, the oxidized Thi and Fc could be electrochemically reduced at two different potentials, and the corresponding electrochemical signals were recorded by differential pulse voltammetry levels. This method achieved the simultaneous determination of two proteins in a single run without obvious nonspecific adsorption and cross-talk.

The in situ transformation of substrates into electroactive products to generate a detection signal is attractive because of the low background signal. Alkaline phosphatese (ALP) has been commonly used in conventional immunoassays to catalyze the hydrolysis of inactive substrates, such as *p*-aminophenyl phosphate (*p*-APP), 1-naphthyl phosphate (1-NP), and *p*-nitrophenyl phosphate (*p*-NPP) [66]. To avoid complicated prior culture and minimal processing steps, Nemr et al. reported a magnetically assisted electrochemical immunosensor for the detection of methicillin-resistant *Staphylococcus aureus* (MRSA) on the basis of a microfluidic device and antibody-functionalized MNPs [67]. As illustrated in Figure 1C, biotinylated rabbit anti-penicillin-binding protein 2a antibodies were modified on the surface of anti-biotin MNPs. MRSA that can express membrane-bound PBP2a was directly captured by the antibody-modified MNPs from nasal swabs without pre-enrichment. After flowing through the microfluidic device under an external magnetic field, the bacteria-modified MNPs were immobilized and then selectively labeled with ALP-labeled signal antibodies. ALP captured by the device could promote the hydrolysis of *p*-aminophenyl phosphate (*p*-APP) into electroactive *p*-aminophenol (*p*-AP). Then, the solution was transferred onto the detection chip to generate an electrochemical signal from the oxidation of *p*-AP. Moreover, the enzymatic products can reduce certain metal ions into electroactive metal on the electrode surface for electrochemical detection. Human epidermal growth factor receptor 2 (HER2) is a specific biomarker for invasive and aggressive breast cancer. Freitas et al. reported a magnetic electrochemical immunoassay of the extracellular domain of HER2 (HER2-ECD) by the enzymatic-product-induced metallization (silver) [68]. As shown in Figure 1D, the HER2-ECD targets were sandwiched between capture-antibody-modified MBs and ALP-labeled signal antibodies. As a result, the captured ALP catalyzed the hydrolysis of the substrate 3-indoxyl phosphate, and the enzymatic product caused the formation of Ag through the reduction of Ag^+^ ions. The oxidation of Ag into AgCl can be readily monitored by linear sweep voltammetry.

#### 2.1.2. Nanomaterials

Nanomaterials with a large surface area can act as nanocarriers for the immobilization of multiple electroactive molecules for signal output and amplification. In this respect, Bu et al. encapsulated Fc molecules into peptide-Cu_3_(PO_4_)_2_ organic–inorganic nanocomposites through a one-step process and used the flower-like nanocomposites as signal reporters to develop a magnetic electrochemical immunosensor for the detection of *Salmonella typhimurium* [69]. As a result, the biosensor was successfully used to determine *Salmonella typhimurium* in milk. Cao et al. reported a MB-based electrochemical immunoassay of tumor marker CD25 through DNA-based signal amplification [70]. After the polycytosine DNA sequence (dC_20_)-modified signal antibodies were immobilized on the electrode through the formation of sandwich immunocomplexes, the phosphate backbone was reacted with molybdate to form molybdophosphate that could be electrochemically reduced.

**Figure 2:**
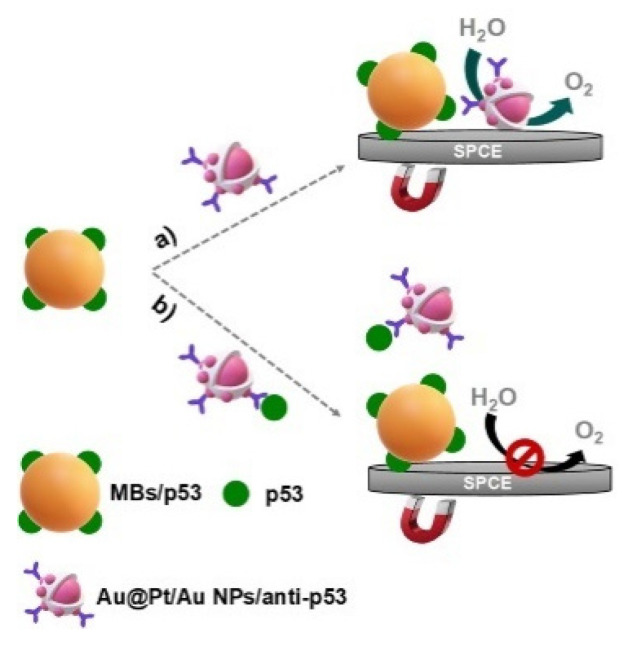
Schematic illustrations of the competitive immunoassay for conformationally altered p53 detection using Au@Pt/Au NP tags. Reproduced with permission [76]. Copyright 2020, American Chemical Society.

Quantum dots (QDs) consisting of abundant metal ions can be utilized as signal tags for electrochemical assays. Freitas et al. reported an immunomagnetic-bead-based method for the detection of breast cancer biomarker HER2-ECD and tumor cells with CdSe@ZnS QDs as the signal labels [71]. In this work, using an acid dissolution process, Cd^2+^ ions were released from QDs and then quantified by differential pulse anodic stripping voltammetry. More interestingly, metal nanomaterials can be utilized in the design of electrochemical biosensors because the metal ion content can be directly determined by simple electrochemical techniques [72,73]. For example, Afonso et al. reported a disposable magneto-immunosensor for *Salmonella* detection using AuNPs as the labels [74]. In this work, AuNPs immobilized on the electrode surface were electrochemically oxidized into Au(III) ions in the presence of HCl. The produced Au(III) could be reduced into Au(0), producing a cathodic signal. In addition, metal nanoparticles can be directly used as the signal labels to catalyze hydrogen evolution in an acidic medium [75]. In this way, Escosura-Muniz et al. developed an electrochemical magnetoimmunosensor for the determination of anti-hepatitis B virus antibodies using AuNPs as the signal labels to catalyze hydrogen evolution in an acidic medium. Iglesias-Mayor et al. reported a competitive immunosensing platform for the detection of conformationally altered p53 peptide using bifunctional Au@Pt/Au NPs as electrocatalytic tags [76]. In this work, the high catalytic Pt and Au protuberances were sequentially grown on the surface of AuNPs through metal depositions and galvanic replacement reactions. The formed Au@Pt/Au NPs could electrochemically catalyze the water oxidation reaction at neutral pH with an increased catalytic activity due to the synergistic effect between Au and Pt. The NPs were further modified with anti-p53 monoclonal antibodies without the inhibition of catalytic capability. Initially, p53-modified MBs brought a large number of Au@Pt NPs to the electrode for water oxidation reaction to generate a high chronoamperometric current (see Figure 2a). Owing to the competitive immunoreactions between p53 analytes and p53-modified magnetic beads to interact with antibody-conjugated Au@Pt NPs, there would be less binding of p53-modified MBs as the analyte concentration increased (see Figure 2b), leading to a decreasing electrochemical signal. Moreover, nanomaterials with enzyme-like activity can also be used to construct magnetically assisted electrochemical immunosensors. For instance, Cao et al. reported an electrochemical immunoassay of carcinoembryonic antigens using antibody-modified Au@Pt nanozymes as the signal probes [77]. In this work, AuNPs and MNP-decorated reduced graphene oxide-tetraethylenepentamine (rGO-TEPA) were modified with capture antibodies (Ab_1_) for the construction of a magnetic sensing platform. The target carcinoembryonic antigen was captured by the antibody-modified magnetic nanocomposites and then modified by the antibody-modified Au@PtNPs. The complexes were magnetically accumulated on the electrode surface. Au@PtNPs in the complexes could accelerate the oxidation of *o*-phenylenediamine into 2,2′-diaminoazobenzene by H_2_O_2_. The produced 2,2′-diaminoazobenzene was electrochemically reduced and the signal change was recorded by square wave voltammetry.

#### 2.1.3. In Situ Assembled DNA Polymers

**Figure 3:**
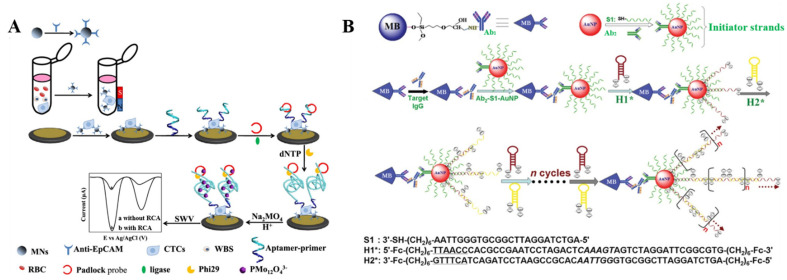
(**A**) Schematic illustration of circulating tumor cell (CTC) measurement in whole blood based on MNP isolation and rolling circle amplification signal amplification. Reproduced with permission [81]. Copyright 2019, American Chemical Society. (**B**) Schematic illustration of the immuno-hybridization chain reaction assay for human IgG detection. Reproduced with permission [82]. Copyright 2012, American Chemical Society.

DNA-based amplification techniques can be integrated with immunoassays for the development of ultrasensitive sensing platforms [78,79,80]. For example, Shen et al. reported an electrochemical biosensor for the detection of circulating tumor cells by integrating magnetic separation, rolling circle amplification, and DNA-generated electrochemical current [81]. As shown in Figure 3A, the breast cancer cell MCF-7 was captured and enriched by anti-epithelial cell adhesion molecule (EpCAM) antibody-modified magnetic nanospheres. Then, the aptamers specifically bound to the MUC1 proteins that were overexpressed on the cell surface. The primer moiety at the end of DNA was hybridized with the circular template in the presence of ligase and then extended through rolling circle amplification reaction with the assistance of DNA polymerase and deoxynucleotide (dNTP). The phosphate backbones of the formed long ssDNA polymer could react with molybdate to form redox molybdophosphate precipitates. The electrochemical signal attributed to the reduction of molybdophosphate was recorded through square wave voltammetry. The biosensor achieved a detection limit of 1 cell/mL for the breast cancer cell MCF-7. Moreover, enzyme-free DNA amplification techniques can also be combined with immunoassays to improve the detection sensitivity. Zhang et al. developed a MB-based immuno-hybridization chain reaction for human IgG detection [82]. As displayed in Figure 3B, monoclonal mouse anti-human IgG (Ab_1_) was immobilized on the MBs through the epoxy–amine reaction. AuNPs were modified with signal antibodies (Ab_2_) and DNA initiators at a high DNA/antibody ratio. In the presence of IgG, the sandwiched immunocomplexes were formed between the antibodies immobilized on the MBs and the signal antibodies on AuNPs. Many DNA initiators could simultaneously trigger multiple hybridization chain reactions in the presence of Fc-labeled haripins H1 and H2. The resulting long nicked double-helix had numerous Fc tags, thus producing a strong electrochemical signal by the oxidation of Fc. The magnetically assisted biosensor based on the immune-hybridization chain reaction showed a low detection limit down to 0.1 fg/mL.

**Table 1:** Analytical performances of magnetically assisted electrochemical immunosensors.

Signal Tags	Targets	Linear Range	Detection Limit	Ref.
HRP	mRNA and IL-8	0.32~7.5 nM for mRNA and 0.0879~5 ng/mL for IL-8	0.21 nM for mRNA and 72.4 pg/mL for IL-8	[49]
HRP	HRP2	0.36~31.3 ng/mL	0.36 ng/mL	[58]
HRP	Ochratoxin A	0.01~0 ppb	0.008 ppb	[59]
HRP	HIF-1α	0.25~10 ng/mL	76 pg/mL	[52]
HRP	5-mC-MGMT and 5-hmC-MGMT	4~250 pM for 5-mC-MGMT and 1.44~100 pM for 5-hmC-MGMT	1.2 pM for 5-mC-MGMT and 0.43 pM for 5-hmC-MGMT	[54]
HRP	Cadherin-17	4.76~1000 ng/mL	1.43 ng/mL	[55]
HRP	miRNA-205	8.2~250 pM	2.4 pM	[51]
HRP	p53-specific autoantibody	1.1~5 U/mL	0.34 U/mL	[57]
Poly-HRP	MMP-9	0.03~2 ng/mL	13 pg/mL	[61]
HRP-AuNPs	CEA	5~60 ng/mL	5 ng/mL	[62]
HRP-Au@Pt-Gr	*Escherichia coli* O157:H7	4.0 × 10^2^~ 4.0 × 10^8^ CFU/mL	91 CFU/mL	[63]
Thi-HRP-SiNPs	CA 125	0.1~450 U/mL	0.1 U/mL	[64]
Thi/Fc-HRP-Fe_3_O_4_-GO	CEA and AFP	0.01~80 and 0.01~200 ng/mL	1 pg/mL	[65]
ALP	MRSA	1 × 10^3^~1 × 10^5^ CFU/mL	845 CFU/mL	[67]
ALP	HER2	5~50 and 50~100 ng/mL	2.8 ng/mL	[68]
Fc@MI-Cu_3_(PO_4_)_2_	*Salmonella typhimurium*	10 to 1 × 10^7^ CFU/mL	3 CFU/mL	[69]
molybdophosphate	CD25	1 pg/mL~1 ng/mL	0.5 pg/mL	[70]
AuNPs	*Salmonella*	1.5 × 10^3^~1.5 × 10^5^ cells/mL	143 cells/mL	[74]
AuNPs	α-HBsAg IgG antibody	5~69.2 mIU/mL	3 mIU/mL	[75]
Au@Pt/Au NPs	p53 peptide	50~1000 nM	66 nM	[76]
Au@Pt NPs	CEA	5 pg/mL~200 ng/mL	1.42 pg/mL	[77]
AuNPs	IgG	0.1 fg/mL~100 ng/mL	0.1 fg/mL	[82]

Abbreviations: HRP, horseradish peroxidase; HRP2, plasmodium falciparum histidine-rich protein 2; HIF-1α, hypoxia-inducible factor-1 alpha; 5-mC, 5-methylcytosine; 5-hmC, 5-hydroxymethylcytosine; MGMT, O-6-methylguanine-DNA methyltransferase; MMP-9, matrix metalloproteinase 9; AuNPs, gold nanoparticles; CEA, carcinoembryonic antigen; Gr, graphene; SiNPs, silica nanoparticles; Thi, thionine; CA, carbohydrate antigen; Fc, ferrocene; GO, graphene oxide; AFP, α-fetoprotein; ALP, alkaline phosphatase; MRSA, methicillin-resistant Staphylococcus aureus; HER2, human epidermal growth factor receptor 2; MI, magainin I; CD25, interleukin-2 receptor alpha chain.

### 2.2. DNA Biosensors

DNA-based electrochemical biosensors have witnessed huge progress because of their remarkable merits of rapid response, easy operation, and high sensitivity [83,84]. The biosensors can easily distinguish complementary and non-complementary DNA or RNA targets through the base-pairing hybridization. Unlike most of the standard DNA-based heterogeneous assays, electrochemical biosensors with DNA-modified MBs exhibited good repeatability and high recognition efficiency due to the low steric hindrance and the high freedom of configuration [85]. DNA can be directly determined by adsorptive stripping voltammetry on the basis of the oxidation of guanosine. In addition, Cu(I)–guanine complexes accumulated on the electrode surface can also generate an electrochemical signal from the oxidation of Cu(I) ions [86]. However, the signals from the electrochemical oxidation of guanosine and Cu(I) are weak, limiting the direct detection of low-level DNA. Thus, electroactive-tag-labeled DNA stands with improved electrochemical signal have been designed and commercialized for the establishment of biosensors. For example, Chen et al. used methylene-blue-labeled DNA-modified gold-coated MNPs to capture and detect circulating tumor DNA through electric-field-induced reconfiguration [87,88]. To improve the sensitivity, several amplification strategies have been integrated with magnetically assisted biosensors to achieve ultrasensitive detection, mainly including enzyme labels, thermal-cycle or isothermal DNA amplification, and functional nanomaterials (Table 2). In addition, multiple strategies based on two or more amplification techniques could further boost the performance and remedy the insufficiency of single amplification.

#### 2.2.1. Enzyme Labels

**Figure 4:**
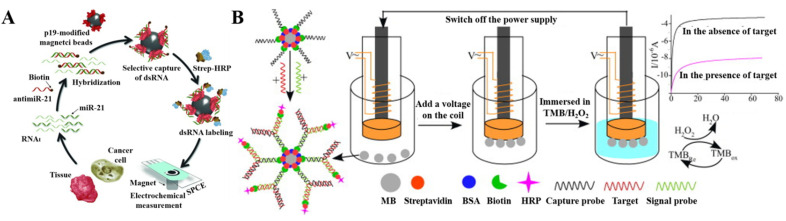
(**A**) Schematic illustration of the p19-based amperometricmagnetosensor designed for the determination of miR-21. Reproduced with permission [91]. Copyright 2014, Wiley. (**B**) Schematic illustration of the magnetic-controllable electrochemical RNA biosensor. Reproduced with permission [94]. Copyright 2013, Elsevier.

Enzymes such as HRP and GOx can be labeled at the end of DNA or RNA as signal reporters. Hybridization between a target probe and a signal probe on the surface of magnetic beads may induce a change of electrochemical signal. Natural enzymes in the hybrids can accelerate the redox or hydrolysis reactions and significantly induce the change of electrochemical signal [89,90,91]. For example, Campuzano et al. reported a HRP-based magneto-biosensor for miR-21 detection [91]. In their design of the biosensor (Figure 4A), the target miR-21 hybridized with biotinylated anti-miR-21 probe to form a duplex. The dsRNA-binding viral protein p19-modified MBs were used to capture the miR-21–antimiR-21 duplex. After the selective capture, the streptavidin (SA)-HRP conjugates were attached to the bead surface through the biotin–SA interactions. HRP catalyzed the reaction between H_2_O_2_ and HQ to generate an electrochemical signal. The reduction current was proportional to the concentration of miR-21. The amperometric biosensors have also been used to determine mitochondrial DNA and methylated DNA with poly-HRP labels [92,93].

On the basis of a template-enhanced hybridization process, the junction-probe strategy has been proposed for DNA detection in which two DNA probes could be brought to each other in the presence of a template target, thus forming a ‘‘Y’’ junction structure. This strategy can improve the detection specificity and sensitivity. In this respect, Wang et al. developed a magnetic-controllable electrochemical RNA biosensor for the detection of hsa-miR-200a that was based on enzymatic amplification and junction-probe strategy [94]. As shown in Figure 4B, the biotin-functionalized capture probe was immobilized on the SA-modified MBs. In presence of the target hsa-miR-200a, a biotin-labeled signal probe was hybridized with both the target and capture probe to form a ternary ‘‘Y’’ junction structure on the surface of MBs. Then, SA-HRP conjugates were captured by the biotinylated signal probes attached on MBs, which were then held by the electrically magnetic-controlled gold working electrode. The H_2_O_2_-meidated oxidation of TMB was enzymatically promoted by HRP, and the continuously generated oxidation products were electrochemically reduced, producing an increased signal.

#### 2.2.2. Thermal-Cycle or Isothermal Amplification

**Figure 5:**
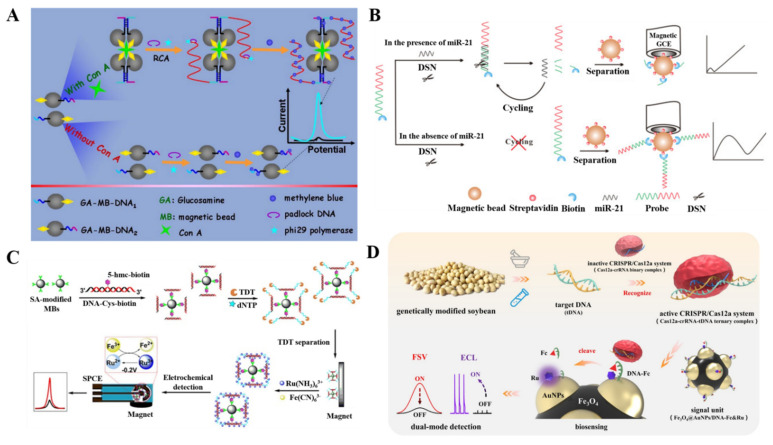
(**A**) Schematic illustration of the proximity ligation assay with three-way junction-induced rolling circle amplification (RCA) for electrochemical detection of concanavalin A (Con A). Reproduced with permission [98]. Copyright 2014, American Chemical Society. (**B**) Schematic illustration of the biosensor for the detection of miRNA-21 based on DSN-assisted target recycling. Reproduced with permission [100]. Copyright 2016, Elsevier. (**C**) Schematic illustration of the label-free and immobilization-free electrochemical magnetobiosensor for 5-hmC assay. Reproduced with permission [101]. Copyright 2019, American Chemical Society. (**D**) Schematic illustration of a CRISPR/Cas12a-mediated dual-mode electrochemical magnetobiosensor for the determination of genetically modified soybean. Reproduced with permission [102]. Copyright 2021, American Chemical Society.

Nucleic acid tool enzymes (e.g., restriction endonuclease, polymerase, and exonuclease)-based target recycling or strand extension and enzyme-free DNA assembly techniques such as hybridization chain reaction and catalytic hairpin assembly are the generally used strategies for signal amplification of DNA biosensors [95,96,97]. Rolling circle amplification, an isothermal amplification process, can generate numerous copies of complementary sequences with the circular template, thus producing a long ssDNA. By coupling proximity ligation assay with three-way junction-induced rolling circle amplification, Liu et al. reported an electrochemical biosensor for concanavalin A detection [98]. As shown in Figure 5A, poly(methacrylic acid)-coated MBs were modified with glucosamine and two different DNA fragments through the carbodiimide coupling reaction. In the presence of concanavalin A, MBs were ligated together on the basis of the interaction between concanavalin A and glucosamine on the bead surface. Under the concanavalin-A-induced proximity ligation assay, a three-way DNA junction formed due to the partial base pairing on the DNA1/DNA2 hybrid. Then, numerous repeated oligonucleotide segments were generated on the MBs through rolling circle amplification under the catalysis of ligase and DNA polymerase. The negatively charged DNA stands could adsorb a large number of positively charged methylene blue molecules, thus producing an amplified electrochemical signal.

As a sequence-independent tool enzyme, exonuclease (Exo) III shows high exodeoxyribonuclease activity for double-strand DNA (dsDNA) in the direction from 3′ to 5′ end, but not ssDNA or dsDNA with a protruding 3′ terminus. In this respect, Li et al. reported a label-free electrochemical magnetic aptasensor for the determination of carcinoembryonic antigen that was based on Exo III-assisted signal amplification [99]. The target carcinoembryonic antigen could bind with its aptamer and retain the signal probe in ssDNA, preventing the hydrolysis of dsDNA by Exo III. Then, the G-quadruplex/hemin complexes were formed with the existence of K^+^ and a voltammetric peak at −0.6 V was observed.

Duplex specific nuclease (DSN) can cleave DNA in dsDNA or DNA/RNA heteroduplexes. The enzyme is partially inactive toward ssDNA, ssRNA, and dsRNA. On the basis of this fact, Zhang et al. reported an immobilization-free electrochemical impedance biosensor for the detection of miRNA-21 that was based on DSN-assisted target recycling [100]. As shown in Figure 5B, in the absence of target miRNA-21, the biotin-labeled DNA capture probes were not hydrolyzed by DSN but could be captured by SA-modified MBs. After the immobilization of DNA-MBs on the magnetic glass carbon electrode, DNA acting as a compact negatively charged layer hampered the electron transfer between the charged redox probe [Fe(CN)_6_]^3−/4−^ and the electrode, leading to a large charge-transfer resistance. However, in the presence of target miRNA-21, the DNA probes could hybridize with the biotin-labeled DNA capture probes to form DNA/miRNA hybrids. The DSN-promoted hydrolysis of DNA in the hybrids caused the release of miRNA-21, initiating the next cycle and resulting in the hydrolysis of numerous DNA probes. Consequently, the amount of DNA on MBs decreased and the formation of negatively charged layer was limited, causing a small charge-transfer resistance.

The terminal deoxynucleotidyl transferase (TDT) can add additional nucleotides at the 3′-end of ssDNA primers without the use of templates. Zhang et al. developed a label-free and immobilize-free magnetobiosensor for the detection of 5-hydroxymethylcytosine (5-hmC) in genomic DNA that was based on TDT enzymatic amplification [101]. As illustrated in Figure 5C, 5-hmC in dsDNA was modified with biotin and then captured by SA-modified MBs. In the presence of TDT and dNTPs, DNA with 3′-OH termini was extended to a long ssDNA. Numerous positively charged Ru(NH_3_)_6_^3+^ ions were adsorbed on the negatively charged DNA via the electrostatic attractions. After the capture of Ru(NH_3_)_6_^3+^-adsorbed MBs by a magnetic electrode, Ru(NH_3_)_6_^3-^ was electrochemically reduced into Ru(NH_3_)_6_^2+^. In this process, the generated Ru(NH_3_)_6_^2+^ was chemically oxidized into Ru(NH_3_)_6_^3+^ by Fe(CN)_6_^3−^, resulting in the recycling of Ru(NH_3_)_6_^3+^ and the generation of an enhanced reduction current.

The CRISPR/Cas system, consisting of the clustered regularly interspaced short palindromic repeats (CRISPR) and CRISPR-associated nucleases proteins (Cas), can be applied in the field of biosensing due to the specific (cis-cleavage) or non-specific nucleic acid degradation ability. Ge et al. reported a CRISPR/Cas12a-mediated dual-mode electrochemical magnetobiosensor for the determination of genetically modified soybean SHZD32-1 [102]. As displayed in Figure 5D, Fe_3_O_4_@AuNPs were used to load Fc-labeled DNA and ruthenium (Ru) complexes through the formation of Au-S and Au-N bonds. The electrochemiluminescence signal from ruthenium complexes was inhibited by Fc through resonance energy transfer. The target DNA (tDNA) could bind with CRISPR-derived RNA (crRNA) and Cas to form a ternary complex. Then, the Fc-labeled DNA on the MBs was cleaved by the complex with non-specific cleavage ability toward ssDNA. The release of Fc molecules resulted in the decrease in the fast scan voltammetric signal and the recovery of the electrochemiluminescent signal from Ru complexes.

**Figure 6:**
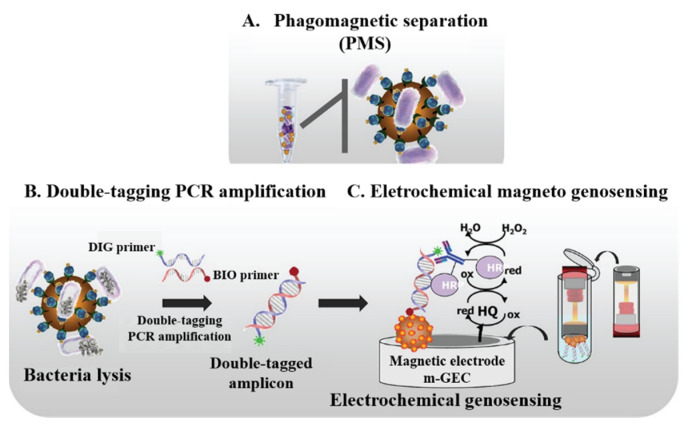
Schematic illustration of the phagomagnetic separation (**A**) of the bacteria followed by the double-tagging PCR (**B**) and the electrochemical magneto-genosensing (**C**). Reproduced with permission [103]. Copyright 2013, American Chemical Society.

DNA-technique-based signal amplification can be integrated with enzyme-based amplification to further improve the sensitivity. Usually, the HRP labels are used to bind with the products of DNA techniques through the biotin–SA or antibody–antigen interactions. Consequently, a large amount of HRP molecules on MBs significantly enhance the electrochemical response. For example, Liebana et al. reported the phagomagnetic separation and electrochemical magneto-genosensing of pathogenic bacteria using HRP–antibody conjugates as the signal tags (Figure 6) [103]. The P22-bacteriophage-modified MBs were used to specifically capture and concentrate the bacteria *Salmonella* by binding to the tail spike proteins on the bacteria surface (Figure 6A). After the lysis of the captured bacteria, the released DNA targets were quantified through the amplification of double-tagging PCR reaction and HRP catalysis (Figure 6B). The double-tagged amplicons were then captured by SA-modified MBs and labeled with HRP-modified antibodies (Figure 6C). After the capture of MB composites by a magnetic electrode, the HRP-catalyzed redox reaction between H_2_O_2_ and HQ produced a strong amperometric signal. Such a signal amplification method was further used to detect *Salmonella* using antibody-modified MBs as the sensing platforms [104].

**Figure 7:**
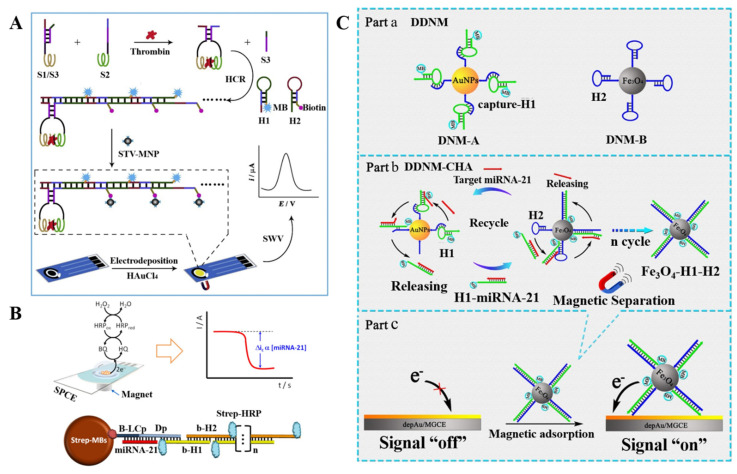
(**A**) Schematic illustrations for the electrochemical thrombin detection method that was based on a target-induced, proximity-binding-induced strand displacement reaction for hybridization chain reaction (HCR) formation of the MNPs/DNA polymers for efficient separation and signal amplification. Reproduced with permission [105]. Copyright 2021, Elsevier. (**B**) Schematic illustration of the amperometric miRNA biosensor designed using MBs as immobilized biomolecule carriers, a sandwich type hybridization assay, HCR amplification, and amperometric detection at disposable electrodes using the HRP/H_2_O_2_/HQ system. Reproduced with permission [106]. Copyright 2012, American Chemical Society. (**C**) Schematic illustrations for (part a) the dual 3D DNA nanomachine (DDNM) consisting of DNM-A and DNM-B, (part b) DDNM-mediated catalytic hairpin assembly (DDNM-CHA), and (part c) construction of an immobilization-free electrochemical biosensor for the detection of miRNA-21. Reproduced with permission [117]. Copyright 2021, American Chemical Society.

Hybridization chain reaction, a simple, isothermal, and enzyme-free reaction process, has become a powerful signal amplification technique for biosensing. Qiu et al. reported an immobilization-free electrochemical strategy for the detection of thrombin that was based on MNP-decorated DNA polymers [105]. As displayed in Figure 7A, the hybridization of the proximity probes S1 and S2 was blocked by probe S3. Thrombin could bind to the aptamer sequences of S1/S3 hybrid and probe S2, decreasing the distance between S1/S3 and S2. Then, probe S1 hybridized with S2, leading to the release of probe S3. The dark red domain of S1 and blue domain of S2 could trigger a hybridization chain reaction in the presence of methylene blue and biotin-labeled hairpin probes H1 and H2. The formed DNA polymers were labeled with SA-modified MNPs and then accumulated on the AuNP-deposited electrode with the aid of a magnet to generate an electrochemical signal from numerous methylene blue molecules. In addition, Torrente-Rodríguez et al. reported the electrochemical determination of miRNA-21 on the basis of the hybridization chain reaction and enzymatic amplification [106]. As displayed in Figure 7B, the target miRNA-21 bound with the initiator strand to initiate the hybridization chain reaction on the surface of MBs. A large number of biotin molecules in the nicked hybridization chain reaction product recruited many SA–HRP conjugates, thus amplifying the electrochemical signal through the HRP-catalyzed reduction of H_2_O_2_ with HQ as the mediator. Moreover, ALP-based amplification has been brought into the hybridization chain reaction system, and the ALP-induced metallization was determined by anodic stripping voltammetry [107]. DNAzymes with enzymatic activity are a type of functional nucleic acids that may be the in-vitro-selected DNA sequences. They retain the advantages of enzymes and DNA and can accelerate different chemical reactions such as redox reaction, DNA and RNA cleavage, ligation, and phosphorylation [108,109]. Some DNAzymes need to be activated by specific species such as metal ions and proteins [110,111]. On the basis of this property, Zhuang et al. reported a DNAzyme-based magneto-controlled electrochemical biosensor for the detection of lead (II) with hybridization chain reaction amplification [112]. In the presence of Pb^2+^, the DNA substrates in the dsDNAzymes were catalytically cleaved. The initiator strands were then captured by MBs to induce the formation of long DNA polymers suspended with numerous Fc molecules, generating a strong electrochemical signal.

Although the DNA-based signal amplification strategies can significantly improve the detection sensitivity, most of them are time consuming and show low conversion efficiency. To address the challenge, nanomaterials were employed as the reaction platforms in the medium to increase the local concentration of reactants and decrease the steric hindrance of solid substrates [113,114,115,116]. Recently, Zhang et al. reported an electrochemical biosensor for the detection of miRNA-21 that was based on a dual 3D DNA nanomachine-based catalytic hairpin assembly [117]. As shown in Figure 7C, AuNPs and MNPs were used as the kernels of two nanomachines for the modification with different DNA strands. MiRNA-21 was able to hybridize with methylene blue-labeled hairpin H1 on AuNPs to form an H1–miRNA duplex that would leave from AuNPs. The exposed ssDNA region in the duplex could hybridize with the toehold of hairpin H2 on MNPs to form an H1–H2 duplex. Then, the released miRNA participated in the next cycle. Eventually, a few miRNA-21 could result in the capture of large amounts of methylene blue-labeled H1 probes on MNPs. After the concentration of modified MNPs by the electrode with a magnet, a strong electrochemical signal was observed from the electrochemical oxidation of methylene blue.

#### 2.2.3. Functional Nanomaterials

**Figure 8:**
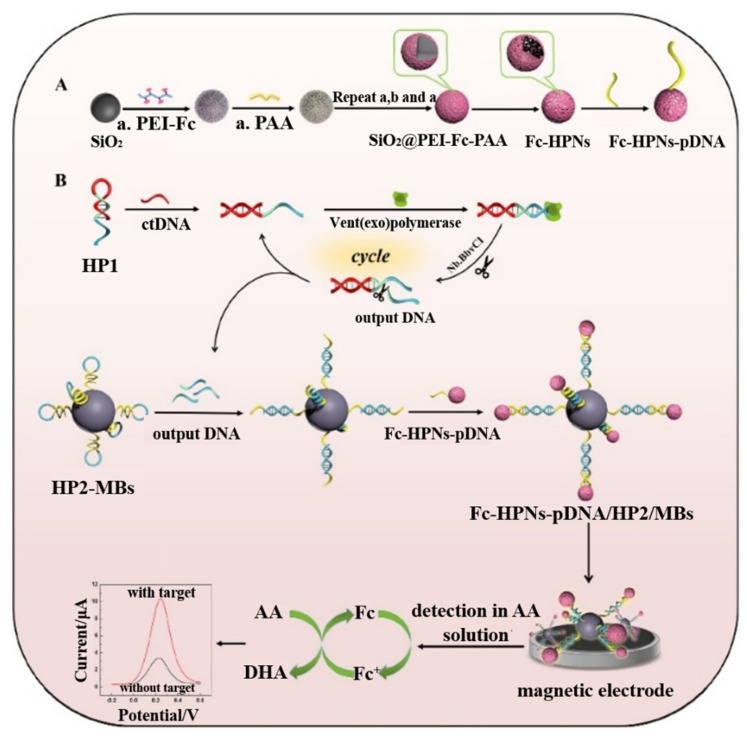
Schematic illustration of (**A**) preparation of the Fc-HPNs-pDNA. (**B**) Design principle of the circulating tumor DNA (ctDNA) biosensor. Reproduced with permission [119]. Copyright 2022, Elsevier.

Nanomaterials with different electrochemical properties can be modified with signal probes and then captured and separated by the complementary DNA-modified MBs. Because of their large surface area and ease of functionalization, nanomaterials have been usually used as nanocarriers for electroactive molecules and DNA probes. For example, Lu et al. used Fc- and SA-modified AuNPs to label biotinylated hairpin DNA probe-coated AuNP magnetic microbeads [118]. Jia et al. reported a magnetically assisted electrochemical biosensor for the detection of circulating tumor DNA that was based on hollow polymeric nanospheres and a dual-enzyme-assisted target amplification strategy [119]. As shown in Figure 8, the Fc-loaded hollow polymeric nanospheres (Fc-HPNs) were prepared through the self-assembly of polyethylenimine-Fc (PEI-Fc) and poly acrylic acid (PAA) on SiO_2_ nanoparticles by hydrofluoric acid etching. The circulating tumor DNA could hybridize with hairpin DNA1 (HP1) to generate numerous output DNAs through the polymerase and nicking endonuclease-based, dual-enzyme-assisted target amplification strategy. Then, the released output DNAs facilitated the capture of pDNA-labeled Fc-HPNs assemble on the MBs through the hybridization reaction. The formed MB conjugates were captured by a magnetic glassy carbon electrode. An enhanced electrochemical signal was produced using ascorbic acid (AA) to catalyze the reduction of Fc molecules. Moreover, metal nanomaterials can be used as the signal tracers to produce an electrochemical signal by stripping voltammetry [120]. For instance, Wang et al. used an indium microrod as the signal tags for the magnetically assisted electrochemical detection of DNA by solid-state derivative chronopotentiometric measurements [121]. Li et al. developed a paper electrochemical biosensor for the detection ofhepatitis B virus DNA using MBs as the mobile solid-phase supports and AgNPs as signal labels [122].

**Table 2:** Analytical performances of magnetically assisted electrochemical DNA biosensors.

Signal Tags	Targets	Linear Range	LOD	Ref.
HRP	5-mC	3.9~500 pM	1.2 pM	[89]
HRP	MTase	0.5~125 U/mL	0.2 U/mL	[90]
HRP	miR-21	0.14~10 nM	0.04 nM	[91]
ProtA-HRP40	dsDNA	0.39~75 pM	0.12 pM	[92]
HRP	miRNA	1 aM~1 fM	0.22 aM	[94]
RCA and methylene blue	Con A	1.96 pM~98 nM	1.5 pM	[98]
G-quadruplex/hemin	CEA	0.1~200 ng/mL	0.4 pg/mL	[99]
DSN-assisted target recycling	miR-21	0.5~40 fM	60 aM	[100]
TDT enzymaticamplification	5-mC	0.01~1000 pM	9.06 fM	[101]
Fc-DNA	SHZD32-1	10~1 × 10^8^ fM	3 fM	[102]
HCR and methylene blue	TB	0.005~50 nM	1.1 pM	[105]
HCR and HRP	miRNA-21	0.2~5 nM	60 pM	[106]
HCR and ALP	miRNA-21	2.5 fM~25 nM	0.12 fM	[107]
DNAzyme, HCR, and methylene blue	Pb^2+^	50 pM~1 μM	15 pM	[110]
DNAzyme, HCR, and Fc	Pb^2+^	0.1~75 nM	37 pM	[112]
CHA and methylene blue	miRNA	0.2 fM~1 nM	0.14 fM	[117]
Fc-AuNPs	miRNA-182	5~100 fM	0.14 fM	[118]
Fc-HPNs	ctDNA	10 fM~10 nM	1.6 fM	[119]
AgNPs	HBV DNA	0~500 pM	85 pM	[122]

Abbreviations: HRP, horseradish peroxidase; 5-mC, 5-methylcytosine; MTase, DNA methyltransferase; RCA, rolling circle amplification; Con A, concanavalin A; CEA, carcinoembryonic antigen; DSN, duplex-specific nuclease; TDT, terminal deoxynucleotidyl transferase; Fc, ferrocene; PCR, polymerase chain reaction; HCR, hybridization chain reaction; TB, thrombin; CHA, catalytic hairpin assembly; ctDNA, circulating tumor DNA; Fc-HPNs, Fc-based hollow polymeric nanospheres.

### 2.3. Aptasensors

Aptamers are a type of synthetic ssDNA or ssRNA that can be screened by the systematic evolution of ligands by exponential enrichment. They can specifically bind to the corresponding targets with high affinity and specificity, including metal ions, small molecules, proteins, cells, and pathogenic microorganisms [123,124,125,126,127]. As promising alternatives to antibodies in bioassays, aptamers show the advantages of low cost, high stability against harsh conditions, and ease of modification. More importantly, the target-binding event can induce a considerable structural change of aptamer or the dissociation of complementary DNA. The released DNA could be further determined by different electrochemical strategies [128,129]. Thus, the combination of aptamers with MBs allows for the design of novel magnetic sensing strategies (Table 3).

**Figure 9:**
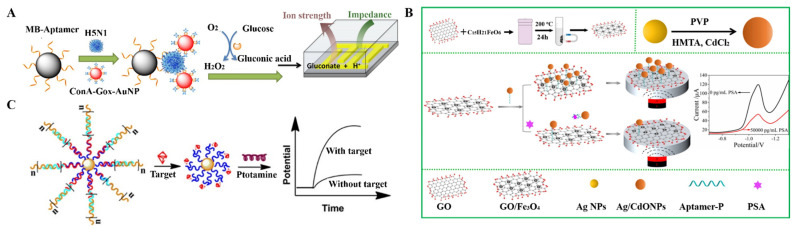
(**A**) Schematic illustration of the biosensing mechanism for avian influenza virus that was based on enzyme catalysis in ultra-low ion strength media using a bare interdigitated electrode. Reproduced with permission [133]. Copyright 2014, American Chemical Society. (**B**) Schematic illustration of the Ag/CdO-NP-engineered magnetic electrochemical aptasensor for prostatic-specific antigen (PSA) detection. Reproduced with permission [135]. Copyright 2019, American Chemical Society. (**C**) Schematic illustration of the DNA-nanostructure-based magnetic beads for potentiometric aptasensing. Reproduced with permission [137]. Copyright 2019, American Chemical Society.

The availability of two aptamers for the target facilitates the design of sandwich-type aptasensors. Usually, one of aptamers is labeled with an enzyme for signal amplification, and the other is modified on the solid surface for target capture. The enzyme label can produce an electrochemical signal with high efficiency and specificity. For example, Centi et al. reported a magnetically assisted aptasensor for electrochemical detection of thrombin that was based on ALP catalysis [130,131]. Amaya-Gonzalez reported the electrochemical competitive assay of hydrophobic protein gluten using HRP to label the aptamer [132]. This method achieved a limit of detection of 0.5 ppb. Fu et al. proposed an impedance sensing method for avian influenza virus detection that was based on enzyme catalysis in ultra-low ion strength media (Figure 9A) [133]. In this work, the biotin-labeled H5Ni aptamer was immobilized on the SA-conjugated MBs, and GOx and Con A were sequentially modified on the AuNPs. The H5N1 virus was captured by the H5N1-specific aptamer-modified MBs and then labeled with Con A and GOx-modified AuNPs through the Con A−glycan interaction. The captured GOx could catalyze the decomposition of glucose, causing an increase in the ion strength and a consequential decrease in the impedance. This method achieved a detection limit of 8 × 10^−4^ hemagglutination units in a 200 μL sample.

Nanomaterials can be modified with an aptamer or its complementary sequence to act as the signal labels. Dou et al. developed an electrochemical aptasensor for circulating tumor cell detection using AuNPs to load different electroactive molecules (Fc and Thi) [134]. Owing to the more specific binding of an aptamer towards its target compared to nanomaterials, Zhao et al. developed a magnetic electrochemical aptasensor for prostatic specific antigen detection (Figure 9B) [135]. In this study, superparamagnetic Fe_3_O_4_/GO nanosheets were adsorbed on aptamer-modified Ag/CdO NPs through hydrophobic and π–π stacking interactions. When the assemblies were attached onto the electrode with the aid of a magnet, the reduction of Cd^2+^ to Cd^0^ in Ag/CdO NPs generated a strong electrochemical peak at −1.07 V. In the presence of a prostatic-specific antigen, the higher affinity between the aptamer and target induced the release of the aptamer-modified Ag/CdO NPs from the surface of Fe_3_O_4_/GO nanosheets, which resulted in the decrease in the reduction current.

The nanostructures formed by various DNA assembly techniques can be used as the signal labels of electrochemical aptasensors [136]. Ding et al. reported a DNA nanostructure-based potentiometric aptasensor for bisphenol A detection [137]. As shown in Figure 9C, the aptamer was immobilized on the surface of MBs. Two auxiliary DNA fragments can initiate a cascade of the hybridization events and lead to the formation of long-range DNA nanostructures. In the presence of bisphenol A, the interaction between aptamer and target resulted in the complete disassembly of the DNA nanostructures. The surface change could be monitored by a polycation-sensitive membrane electrode with protamine as the indicator.

**Figure 10:**
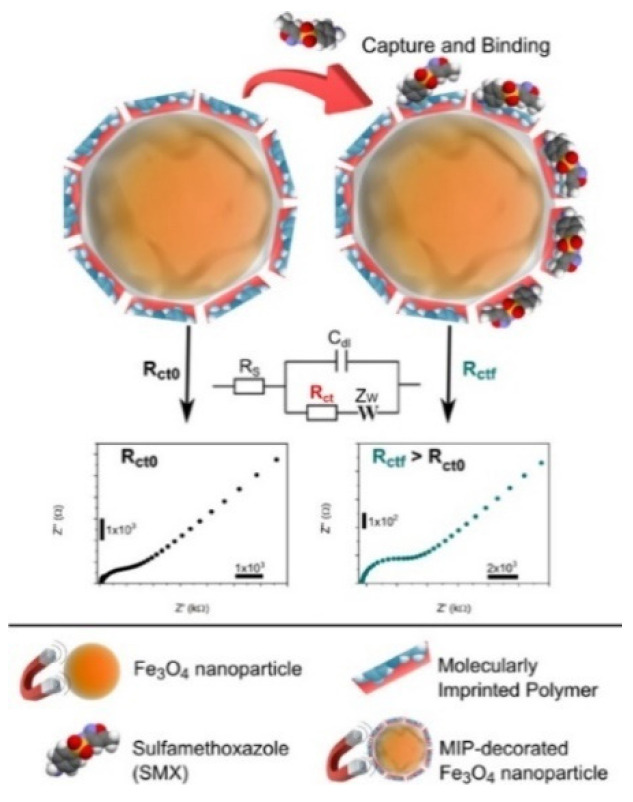
Schematic illustration of the magnetic MIPs for selective and label-free detection of SMX. Reproduced with permission [141]. Copyright 2016, American Chemical Society.

Molecularly imprinted polymers (MIPs) can be used as biomimetic recognition elements (artificial receptors) for the construction of electrochemical biosensors [138,139,140]. Compared with the above-mentioned recognition elements, MIP-based molecular recognition shows the advantages of high stability, low cost, an animal-free nature, and large-scale production. Magneto-actuated molecularly imprinted polymers (magnetic-MIPs), consisting of the molecular imprinting surface as the shell and a paramagnetic core, can integrate the synergic advantages of MIPs and MPs. In this respect, Zamora-Galvez et al. reported a magnetic MIP for selective and label-free detection of sulfamethoxazole (SMX) (Figure 10) [141]. In this work, SMX molecules blocked the electron transfer between the electrode surface and the solution by binding with the recognition sites or cavities of the composites, thus leading to an increase in the charge-transfer resistance.

**Table 3:** Analytical performances of magnetically assisted electrochemical aptasensors.

Signal Tags	Targets	Linear Range	LOD	Ref.
ALP	Thrombin	0~100 nM	0.45 nM	[130]
HRP	Gliadin	0.1 ppb~10 ppm	0.5 ppb	[132]
Fc- and Thi-labeled AuNPs	Ramos cells and CCRF-CEM	5~500 cells/mL	4 and 3 cells/mL	[134]
Ag/CdO NPs	PSA	0.05~50 ng/mL	28 pg/mL	[135]
DNA nanostructures	Bisphenol A	0.1~100 nM	80 pM	[137]

Abbreviations: ALP, alkaline phosphatase; HRP, horseradish peroxidase; GOx, glucose oxidase; CCRF-CEM, human acute lymphatic leukemia cells; PSA, prostatic-specific antigen.

## 3. MB/MNP-Based Target Conversion

The recognition reaction on a nanoparticle surface in a homogenous solution exhibited a faster kinetic in contrast to that on a solid electrode surface. Furthermore, the target detection can be converted into the assay of other species, which could be coupled to different signal amplification strategies for indirect detection of targets [142,143]. In general, the sensing principle of magnetic-assisted electrochemical biosensors relying on the MB/MNP-based target conversion can be divided into stimuli–response release of signal reporters, enzymatic production of electroactive species, and target-induced generation of messenger DNA (Table 4).

### 3.1. Stimuli–Response Release of Signal Reporters

**Figure 11:**
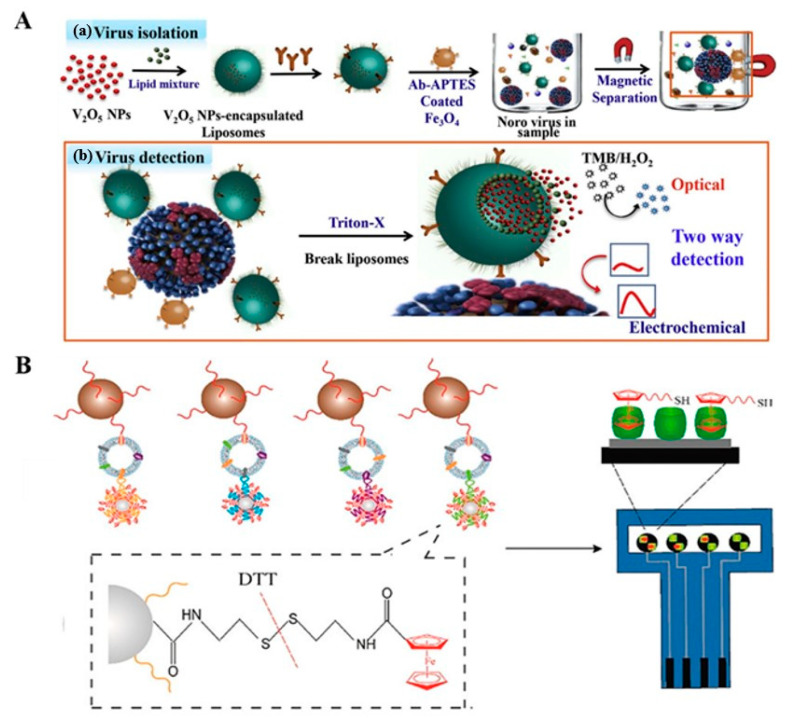
(**A**) Schematic illustrations for the preparation process of V_2_O_5_-NP-encapsulated liposomes (a) and norovirus detection principle (b). Reproduced with permission [147]. Copyright 2020, Elsevier. (**B**) Schematic illustrations for the magneto-mediated electrochemical sensor for exosomal proteins analysis based on host−guest recognition. Reproduced with permission [148]. Copyright 2020, American Chemical Society.

Porous or hollow nanomaterials such as mesoporous silica nanoparticles and metal organic frameworks are the generally used stimuli-responsive nanocarriers to encapsulate electroactive molecules and nanoparticles because of their large surface area, tunable pore size, and excellent porosity [144,145,146]. After immunoreaction and separation, a certain stimulus can be introduced to trigger the release of abundant guests, which can be sensitively determined by the modified electrode. For example, Ganganboina et al. proposed a dual modality sensor for norovirus detection by the liposome-based signal amplification [147]. As displayed in Figure 11A, V_2_O_5_ NPs as nanozymes were encapsulated into liposomes due to their unique advantages of immense catalytic ability, robust stability, and facile surface reformation, followed by functionalization with norovirus-specific antibodies. After the magnetic separation and recognition in the medium, V_2_O_5_ NPs were released to act as peroxidase mimics and electrochemical redox indicators for the generation of colorimetric and robust electrochemical dual signals. In addition, An et al. reported a magneto-mediated electrochemical aptasensor for simultaneous detection of breast cancer exosomal proteins using SiO_2_ NPs to load the signal molecules of N-(2-((2-aminoethyl)disulfanyl)ethyl) ferrocene carboxamide (FcNHSSNH_2_) [148]. As shown in Figure 11B, SiO_2_ NPs were modified with the aptamers of MUC1, HER2, EpCAM, and carcinoembryonic antigen, and then were loaded with FcNHSSNH_2_ molecules on the basis of the reaction between the amino groups of aptamers as well as FcNHSSNH_2_ and the aldehyde groups on the SiO_2_ NPs. The CD63-aptamer-modified MBs were used to capture exosomes. After that, four different aptamer-modified SiO_2_ NPs were added to identify the corresponding exosomal proteins. In the presence of dithiothreitol, FcNHSSNH_2_ molecules were released and detected by the graphene oxide-cucurbit-modified [7] screen-printed carbon electrode. In addition, Hou et al. developed an electrochemical cytosensor by monitoring the release of DNA from circulating tumor cells [149]. The released DNA could react with molybdate to form electroactive molybdophosphate.

**Figure 12:**
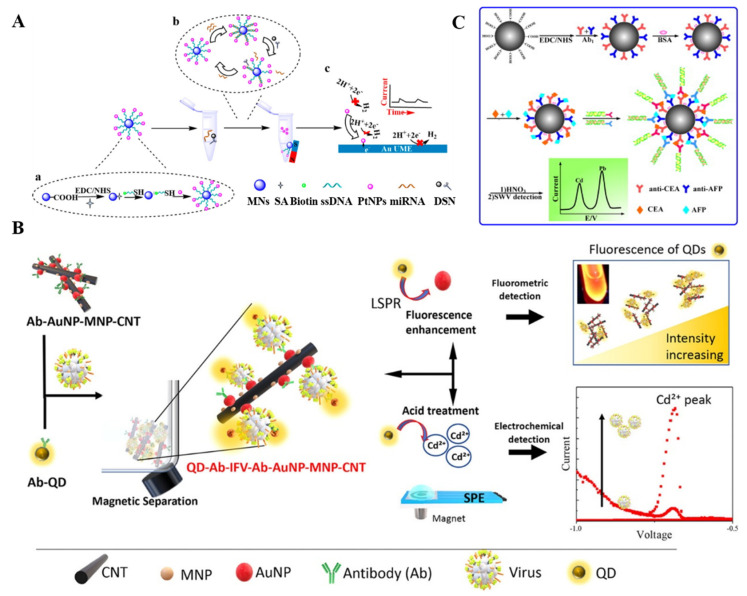
(**A**) Schematic illustrations for the one-to-many single-entity electrochemistry biosensing using satellite MN-DNA-Pt NP conjugates. Reproduced with permission [150]. Copyright 2020, American Chemical Society. (**B**) Schematic illustration of the optical and electrochemical virus detection method using plasmon nanocomposites and QDs. Reproduced with permission [159]. Copyright 2021, American Chemical Society. (**C**) Schematic illustration of the electrochemical immunosensor for the simultaneous detection of carcinoembryonic antigen (CEA) and α-fetoprotein (AFP) that were based on QDs/DNA nanochains as labels. Reproduced with permission [165]. Copyright 2013, Elsevier.

Nanomaterials with electrocatalytic activity can be used to amplify the electrochemical signal. In single-entity electrochemistry, the stochastic collision of a single entity (e.g., nanoparticle, cell, and molecule) on the surface of ultramicroelectrode could generate discrete electrochemical responses. Bai et al. reported a “one-to-many” single-entity electrochemical biosensor for the detection of miRNA-21 that was based on DSN-assisted target-recycling and Pt-NP-based signal amplification [150]. As displayed in Figure 12A, Pt NPs were conjugated on the ssDNA-modified MNPs to form the satellite composites. The target RNA could hybridize with ssDNA on the MBs, and NSN could cleave the DNA in the duplex. Then, the released target RNA hybridized with other ssDNA stands, initiating the DSN-assisted target recycling amplification. Finally, many nearly naked Pt NPs were released to collide effectively on Au ultramicroelectrode (UME), thus generating a reduction current. On the basis of this sensing principle, Luo et al. achieved the detection of HIV-DNA by combining Pt NP-based single-entity electrochemical biosensing with a DNA walker [151].

Nanomaterials such as semiconductor QDs and metal nanoparticles are composed of many metal ions, which can be introduced into the design of magnetically assisted electrochemical biosensors [152,153,154]. For instance, Wang et al. demonstrated that the colloidal gold tags captured by DNA-modified MBs could be dissolved under an acidic environment, and the released Au(III) could be determined by anodic stripping voltammetry [155]. In this way, the authors developed an AuNP- and MB-based biosensor for the detection of DNA hybridization. Liu et al. reported an electrochemical biosensor for the quantification of single-nucleotide polymorphisms using cadmium-phosphate-loaded apoferritin nanoparticles as signal probes [156]. This has allowed the authors to accurately determine single-nucleotide polymorphisms. Wang et al. developed a magnetic electrochemical immunoassay for the detection of phosphorylated acetylcholinesterase by quantifying the content of released Cd^2+^ ions from CdS@ZnS QDs by square wave voltammetry [157]. Li et al. presented a dual-amplified electrochemical biosensor for mRNA detection with DSN and QD-based bio-barcode conjugates [158]. In their work, the capture DNA-modified MBs were linked to the CdS QD-labeled AuNPs. In the presence of mRNA, the dsDNA sandwiched between the capture DNA and mRNA was cleaved with the aid of DSN. Many CdS-QD-based bio-barcode conjugates were released into the solution. Then, the CdS QDs were dissolved with HNO_3_ and the released Cd^2+^ ions were detected by anodic stripping voltammetry. The oxidation current was proportional to the concentration of mRNA. Recently, Takemura et al. developed a plasmon-nanocomposite-enhanced optical and electrochemical immunosensor for the detection of viruses [159]. As shown in Figure 12B, the antibody-modified AuNP-MNP-carbon nanotube (Ab-AuNP-MNP-CNT) nanocomposites were utilized for the magnetic separation of virus. The captured virus could be labeled with antibody-modified CdSeTeS QDs and then separated by a magnet. The fluorescence of QDs was enhanced by the localized surface plasmon resonance of AuNPs, which could be detected by fluorometry. In the electrochemical detection mode, a large number of Cd^2+^ ions released from QDs were detected by differential pulse voltammetry. Moreover, other nanomaterials such as polystyrene nanoparticles, silica nanoparticles, and graphene can also be employed to carry the QD labels for signal amplification [160,161,162]. On the basis of the well-resolved electrochemical signals of metal ions at different potentials, ZnS, CdS, PbS, and CuS QDs have been used as the tracers to develop coding bioassays for the simultaneous electrochemical detection of multiple targets, including DNA and proteins [163,164]. In addition, Kong et al. reported an electrochemical immunosensor for the simultaneous detection of carcinoembryonic antigen and α-fetoprotein with QD/DNA nanochains as signal labels (Figure 12C) [165]. In this work, the carboxyl-functionalized MBs were modified with two different antibodies and then blocked with bovine serum albumin (BSA). The DNA stands were used as the carriers to load CdS and PbS QDs and then labeled with different antibodies. After the capture of carcinoembryonic antigen and α-fetoprotein by antibody-modified MBs, the CdS/DNA- and PbS/DNA-modified antibodies were added to specifically label the corresponding antigens on MBs. A large amount of metal ions with different redox potentials were then released via the acid-dissolution process, which could be determined by square wave stripping voltammetry. The oxidation currents were proportional to the concentration of corresponding proteins.

Besides QDs, metal nanoparticles can also be used as signal tags by the release of metal ions in an acidic medium, and the signal was monitored by differential pulse voltammetry. For example, Abbaspour et al. reported a dual-aptamer-based sandwich detection of *Staphylococcus aureus*, in which the aptamer-modified MBs were used to capture the target and aptamer-conjugated signal reporters of silver nanoparticles [166]. Cao et al. reported the synthesis of copper nanoparticles with DNA as the template and then achieved the electrochemical detection of breast cancer cells using anti-CD44-protein-functionalized MBs to capture the cells and DNA-templated copper nanoparticles [167]. In these reports, the released Ag^+^ and Cu^2+^ ions were quantified by differential pulse voltammetry.

### 3.2. Enzymatic Production of Electroactive Species

**Figure 13:**
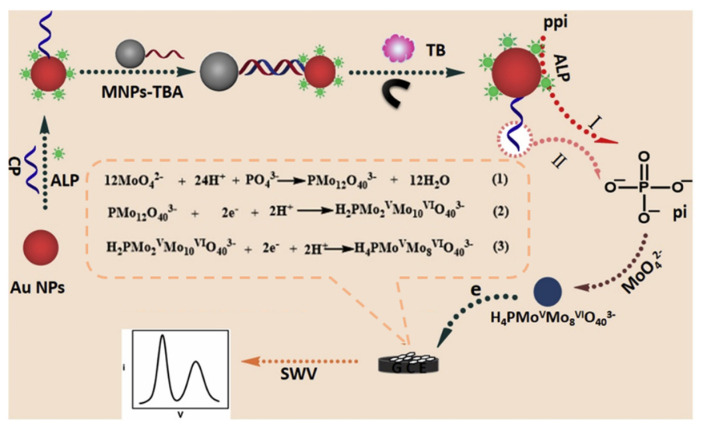
Schematic illustrations for the DNA synergistic enzyme-mediated cascade reaction for homogeneous electrochemical bioassay. Reproduced with permission [172]. Copyright 2019, Elsevier.

The sensitivity of magnetically assisted electrochemical biosensors can be improved through the enzymatic cascade amplification strategy. ALP can catalyze the hydrolysis of inactive substrates into electroactive products. The resulting products could be directly determined by electrochemical oxidation [168,169,170]. Dequaire et al. reported a competitive immunoassay of 2,4-dichlorophenoxyacetic acid using nafion-film-coated screen-printed electrode to detect the ALP product [171]. This method showed a detection limit of 0.01 μg/L and successfully achieved the detection of 2,4-dichlorophenoxyacetic acid spiked in river water samples. Zhang et al. reported a homogeneous electrochemical bioassay of thrombin (TB) that was based on DNA-synergistic-enzyme-mediated cascade reaction (Figure 13) [172]. In this work, AuNPs were labeled with DNA and ALP and then tethered on MBs through hybridization reaction. In the presence of analytes, the DNA/ALP-labeled AuNPs were released from MBs and retained in the supernatant. After the addition of pyrophosphate ions and molybdate, phosphate ions were generated through the ALP-catalyzed hydrolysis. The phosphate groups in the DNA backbones were then reacted with molybdate to produce redox precipitates on the surface of the reduced graphene-oxide-modified electrode, thus producing a strong electrochemical signal quantitatively related to thrombin concentration.

### 3.3. Target-Induced Generation of Messenger DNA

Trace target can trigger the generation of abundant messenger DNA probes through magnetically assisted target conversion. The produced messenger DNA could be determined by different electrochemical strategies [173]. For example, on the basis of the DNA assembly reaction on the electrode, Yang et al. designed a protein-converting strategy for electrochemical detection of the protein cystatin C [174]. In this work, the presence of cystatin C caused the release of abundant output S1 probes through immunoreaction-induced DNA strand displacement reaction and T7 exonuclease (T7 Exo)-assisted protein cyclic enzymatic amplification. The S1 DNA triggered the hybridization chain reaction on the electrode and facilitated the attachment of abundant electroactive Thi molecules that would yield a voltammetric signal. The electrochemical biosensor exhibited a linear range from 0.01 pg/mL to 30 ng/mL with a low detection limit (3 fg/mL). Dong et al. reported an electrochemical aptasensor for tumor exosome detection based on Exo III-assisted cyclic amplification [175]. In this study, the target-induced release of DNA accelerated the hydrolysis of numerous DNA probes immobilized on the electrode by Exo III-assisted cyclic amplification. Thus, the amount of Ru(NH_3_)_6_^3+^ ions adsorbed on the negatively charged DNA significantly decreased, alongside the decrease in the peak current of Ru(NH_3_)_6_^3+^.

**Figure 14:**
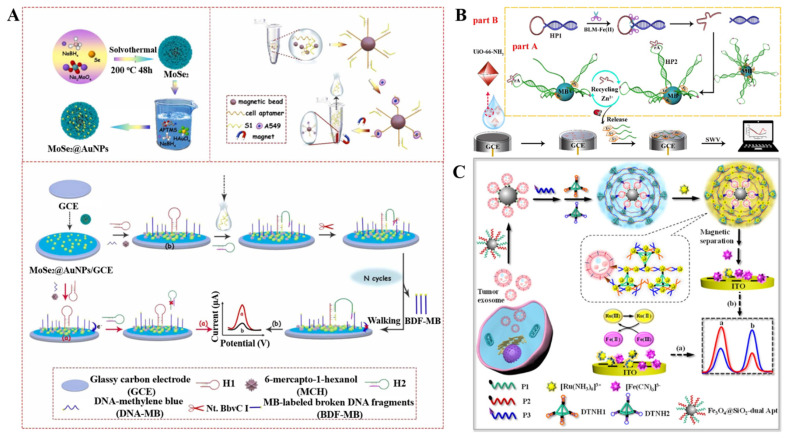
(**A**) Schematic illustrations for the DNA walker induced a “signal off” electrochemical sensor for tumor cells detection. Reproduced with permission [178]. Copyright 2022, Elsevier. (**B**) Schematic illustrations for the BLM assay based on BLM-mediated activation of Zn^2+^-dependent DNAzyme for the release of massive Fc-DNAs and preparation of MOF/GCE and the successive adsorption of Fc-DNAs by MOF/GCE for electrochemical measurement. Reproduced with permission [181]. Copyright 2021, Elsevier. (**C**) Schematic illustration of the ratiometric immobilization-free electrochemical sensing system for tumor exosome detection. Reproduced with permission [184]. Copyright 2021, American Chemical Society.

DNA walker, a typical dynamic DNA device, has gained extensive attention in the field of biosensing of nucleic acids, proteins, and cells. It contains a walking strand initially bound to an underneath track through base-pairing with anchor strands. Under a certain form of energy input as the driving force, continuous locomotion of the walker is realized along a designed trajectory on the surface of electrode or nanoparticle, thus amplifying the signal [176]. Chai et al. developed a bipedal DNA walker-based electrochemical sensing strategy for DNA detection [177]. In this work, the target induced the release of a large number of DNA sequences through the polymerase and nicking endonuclease-assisted target recycling and strand displacement reaction. Then, the bipedal DNA walking on the electrode surface occurred, and the AgNPs were immobilized on the electrode by interacting with the amino groups of the DNA walkers. The captured AgNPs can produce a well-defined silver stripping peak in an aqueous KCl electrolyte medium through the highly characteristic solid-state Ag/AgCl redox process. Recently, Wang et al. developed a DNA walker-induced “signal-off” electrochemical cytosensor for the detection of tumor cells (Figure 14A) [178]. In this study, MoSe_2_@AuNPs nanomaterials prepared by hydrothermal synthesis were used to modify the electrode for the immobilization of hairpin probe 1 (H1) and methylene-blue-labeled DNA. The amine-terminated cell aptamers immobilized on the carboxyl-modified MBs by amide bonds hybridized with ssDNA (S1) probes. The target A549 cells preferentially bound to the aptamers with the high affinity, thus inducing the release of S1 probes from MBs. After the magnetic separation, the released S1 probes in supernatant hybridized with H1 probes on the electrode. The exposed tail sequence in the probe could hybridize with hairpin probe 2 (H2). Meanwhile, the exposed tail sequence of H2 acting as the swing arm of the DNA walker hybridized with methylene-blue-labeled DNA on the electrode. The formed duplex between H2 and methylene-blue-labeled DNA was cleaved by endonuclease Nt.BbvC I, leading to the release of methylene-blue-labeled DNA. The released swing arm could hybridize with other methylene-blue-labeled DNA to trigger a new round of cleavage reaction, realizing the enzyme-assisted cycle amplification. Finally, many methylene-blue-labeled DNA fragments were released from the electrode surface, leading to a decrease in the electrochemical signal.

In most electrochemical biosensors, the sequential immobilization process is tedious and time-consuming, which leads to low reproducibility and fragile stability. Thus, immobilization-free electrochemical biosensors with high simplicity and low cost and robust assays are promising for batch and in-field applications. The functionalized electrode can be used to recruit the released DNA species without the immobilization process [179,180]. For example, He et al. reported an electrochemical biosensor for bleomycin detection using a MOF-modified electrode to concentrate the released DNA (Figure 14B) [181]. In this method, target bleomycin interacted with Fe(II) to form the bleomycin -Fe(II) complex that could reduce oxygen to free radicals and induce the cleavage of hairpin probe DNA1 (HP1) at the recognition site (5′-GC-3′) in the stem. The released DNA acting as DNAzyme fragment could trigger the secondary cleavage of hairpin probe DNA2 (HP2) on MBs with the aid of Zn^2+^-dependent DNAzyme. Then, the DNAzyme fragment was released again and induced the hydrolysis of other HP2 probes on MBs. Finally, abundant Fc-labeled DNA fragments were released and adsorbed onto the MOF-modified electrode, producing a strong electrochemical signal. Meanwhile, Sha et al. used a cucurbit [7] uril (a macrocyclic molecule)-modified electrode to capture the released methylene blue tags through the host–guest interactions [182]. In this work, programmed-death-1-expressing exosomes were first captured by anti-programmed death-ligand 1 antibody-modified MBs and then labeled with cholesterol-modified hairpin templates. After the programmable DNA synthesis and CRISPR-Cas12a-catalyzed random cleavage, methylene-blue-labeled signal strands were cleaved. The released methylene blue tags were captured by the cucurbit [7] uril-modified electrode, producing a strong electrochemical signal.

In recent years, label-free homogeneous electrochemical biosensors have been widely developed because of their low cost and easy preparation [183]. Conventionally, electroactive molecules will readily diffuse towards an electrode surface to undergo an electron transfer reaction and produce an electrochemical signal. In contrast, the intercalation of these molecules into G-quadruplex DNA or dsDNA may limit their diffusion, thus leading to a decrease in electrochemical signal. On the basis of this concept, Yang et al. developed a label-free and immobilization-free ratiometric electrochemical biosensor for the quantification of tumor exosomes [184]. As shown in Figure 14C, the carboxyl-functionalized MUC1 aptamer (P1) and CD63 aptamer (P2) were simultaneously immobilized on the surface of amino-modified Fe_3_O_4_@SiO_2_ NPs. Tumor exosomes that can overexpress MUC1 and CD63 proteins on their surface were captured by the dual-aptamer-modified Fe_3_O_4_@SiO_2_ NPs through the aptamer–target interactions. Then, the cholesterol-modified DNA probe (P3) was tethered to the surface of exosomes on the basis of the hydrophobic effect between cholesterol moieties and the phospholipid bilayer of exosomes. The DNA immobilized on the exosome surface could trigger the hyperbranched hybridization chain reaction in the presence of two-hairpin-functionalized DNA tetrahedral nanostructures. The formed MBs−exosome−hyperbranched DNA superstructures could extract abundant Ru(NH_3_)_6_^2+^ ions, thus leading to the decrease in the amount of Ru(NH_3_)_6_^2+^ in solution. As a result, the redox reaction between electroactive [Fe(CN)_6_]^3−^ and Ru(NH_3_)_6_^2+^ was suppressed, and the *I*[Fe(CN)_6_]^3−^/IRu(NH_3_)_6_^2+^ value was increased.

### 3.4. Other Biosensors with MB/MNP-Based Target Conversion

**Figure 15:**
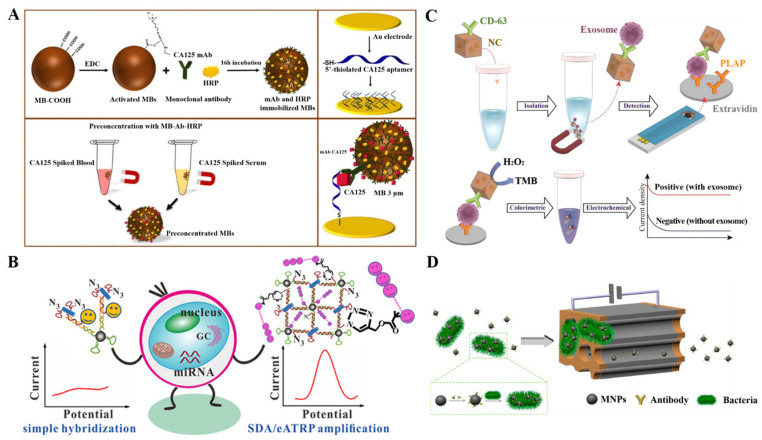
(**A**) Schematic illustration of the dual-bioreceptor immunoassay construction. Reproduced with permission [189]. Copyright 2020, Elsevier. (**B**) Schematic illustration of an enzyme-free electrochemical platform for miRNA-21 detection that was based on MBs and de novo growth of electroactive polymers. Reproduced with permission [196]. Copyright 2021, American Chemical Society. (**C**) Schematic illustration of the nanozyme-based assay for direct exosome isolation and detection from cell culture media. Reproduced with permission [197]. Copyright 2021, American Chemical Society. (**D**) Schematic illustration of the integrated nanochannel-electrode-based separation-detection system for the detection of bacteria. Reproduced with permission [200]. Copyright 2020, American Chemical Society.

Acceptor-modified MBs used to capture and separate the target can be captured by a well-designed modified electrode [185]. This method can suppress electrode biofouling from interfering proteins in biofluids. For this consideration, Premaratne et al. demonstrated that the adsorption of MBs on the electrode through the antibody–antigen interaction could increase the resistance to the redox probe in solution [186]. Enzymes and antibodies can be simultaneously immobilized on the MBs for pre-concentration of target and signal readout [187,188]. Sadasivam et al. reported a magnetically assisted electrochemical detection of the carbohydrate antigen CA125 with enzyme- and antibody-modified MBs [189]. As shown in Figure 15A, MBs were modified with monoclonal antibodies and HRP molecules. After the recognition reaction, the target–MB complexes were immobilized on the aptamer-modified electrode. Then, the HRP labels catalyzed the redox reaction between H_2_O_2_ and DSN, which could be measured by cyclic voltammetry and chronoamperomtry. Wu et al. used polyclonal antibody and ALP-modified MNPs as the signal tag to extract the target virus [190]. After the isolation of H7N9 avian influenza virus, the modified MNPs were captured by the antibodies immobilized on the microelectrode arrays. ALP on MNPs could catalyze the hydrolysis of *p*-APP into reductive *p*-AP. The resulting *p*-AP could reduce Ag^+^ ions to Ag^0^ that are deposited on microelectrode surface. The content of deposited Ag^0^ could be detected by linear sweep voltammetry, which was linearly proportional to the concentration of virus. Moreover, microfluidic techniques integrated with nanostructured electrode arrays and magnetic separation have also been used to detect multiplexed targets [191]. Malhotra et al. developed a nanostructured microfluidic array for the detection of proteins using antibody and HRP-modified MBs [192]. In this study, the targets were captured by MBs, and the resulting magnetic composites were injected into the antibody-covered arrays. Then, the HRP-catalyzed reaction between H_2_O_2_ and HQ enhanced the electrochemical signal. Fapyane et al. reported an electrochemical biosensor for the determination of synthetic DNA and cell-isolated RNA using DNA and cellulase-labeled MBs [193]. In this work, after the capture and deposition, cellulase on MBs could digest the insulating nitrocellulose films on the electrode and thus enhance the electrochemical response.

Nanometarials can be used as bridges to increase the loading of signal reporters. Zhao et al. employed antibody-modified MNPs to capture target and methylene-blue-labeled aptamer-modified AuNPs [194]. The formed magnetic gold nanocomposites were tethered onto the electrode surface through the hybridization reaction, thus producing an amplified signal from methylene blue. Lu et al. used MBs to load Fc-labeled DNA-modified AuNPs as the platform for the DNA walkers [195]. Peng et al. developed an enzyme-free magnetic electrochemical platform for miRNA-21 detection that was based on the de novo growth of electroactive polymers [196]. As shown in Figure 15B, the target miRNA-21 induced the hybridization of hairpin DNA1 on MBs (MBs-H1) and hairpin DNA2 modified on the gold nanorods (AuNRs-H2) through the SDA reaction. Finally, the DNA network structures formed by the assembly of multiple MB-H1/AuNR-H2 composites were obtained. The eATRP initiator, propargyl-2-bromoisobutyrate (PBIB), was conjugated to the 3′-terminus of H2 by the click reaction, which triggered the de novo growth of a number of electroactive polymers with ferrocenylmethyl methacrylate (FMMA) monomers, thus generating an amplified electrochemical signal.

Magnetic Fe_3_O_4_ NPs with HRP-like activity can be used as nanozymes to develop various biosensors. Recently, Boriachek et al. developed a simple method for direct isolation and detection of exosomes using AuNP-loaded nanoporous ferric oxide nanocubes (Au-NPFe_2_O_3_NC) nanozymes [197]. As shown in Figure 15C, Au-NPFe_2_O_3_NC modified with CD63 antibodies were dispersed in sample fluids to capture exosomes. After magnetic separation, the Au-NPFe_2_O_3_NC-bound exosomes were transferred onto the PLAP antibody-modified screen-printed carbon electrode. The Au-NPFe_2_O_3_NC could catalyze the oxidation of TMB in the presence of H_2_O_2_, thus generating an amperometric signal.

Artificial solid-state nanochannels have been extensively explored in the fields of molecular filtration and biosensing because of their merits of mechanical and chemical stability, tunable channel shape, and tailorable surface properties [198,199]. Zhu et al. designed a separation detection system for the detection of *Salmonella* that was based on the larger size of the MB–target complex [200]. As shown in Figure 15D, the outer surface of the anodic aluminum oxide nanochannel array was deposited with a gold electrode layer. After the capture and separation, free and small-sized MNP-Ab would pass through the nanochannel, leaving the larger MNP-Ab-*Salmonella* on the electrode surface. Then, electrochemical impedance spectroscopy was used to measure the signal on the basis of the hindrance of electron transfer on the electrode by MNP-Ab-*Salmonella*.

**Table 4:** Analytical performances of magnetically assisted homogeneous electrochemical biosensors.

Signal Probes	Targets	Linear Range	LOD	Ref.
V_2_O_5_-NP-encapsulated LPs	norovirus	0.01~10pg/mL	4.1 fg/mL	[147]
Fc-labeled SiO_2_ NPs	exosomes	1.2 × 10^3^~1.2 × 10^7^ particles/μL	Not reported	[148]
Molybdophosphate	MCF-7 cell	5~1000 cells/mL	2 cells/mL	[149]
PtNPs	miRNA-21	50 aM~5 nM	47 aM	[150]
PtNPs	HIV-DNA	5 fM~50 nM	4.86 fM	[151]
QDs	BChE	0.1~20 nM	0.05 nM	[153]
HRP-AuNPs	IgG	2.5 × 10^−6^~1µg/mL	260 pg/mL	[154]
QDs	OP-AChE	0.3~300 ng/mL	0.15 ng/mL	[157]
CdS QDs	mRNA	1 fM~0.1 nM	0.48 fM	[158]
CdSeTeS QDs	influenza virus A	1 fg/mL~1 μg/mL	13.66 fg/mL	[159]
QD-PS beads	DNA	0.5 fM~10 pM	0.22 fM	[160]
CdTe or PbS QDs-coated silica	OTA and FB1	0.01~ 10 ng/mL and 0.05~50 ng/mL	5 and 20 pg/mL	[161]
CdS and PbS QDs	CEA and AFP	0.1~100 ng/mL and 0.5~200 ng/mL	3.3 and 7.8 pg/mL	[165]
AgNPs-aptamer	*S. aureus*	10~1 × 10^6^ CFU/mL	1 CFU/mL	[166]
CuNPs-DNA	CD24+ cells	1 × 10^2^~5 × 10^6^ cells	42 cells	[167]
HAP	thrombin	0.1 fM~1 nM	0.40 fM	[169]
ALP-AuNPs	thrombin	1 fM~10 nM	0.26 fM	[172]
dsDNA polymers and Thi	cystatin C	10 fg/mL~30 ng/mL	3 fg/mL	[174]
Exo III-assisted recycling	exosomes	1 × 10^3^~1.2 × 10^5^ particles/μL	70 particles/μL	[175]
CHA	Cu^2+^	1 pM~500 nM	0.33 pM	[176]
AgNPs	DNA	1~100 fM	0.22 fM	[177]
DNA walker	A549 cells	5~5 × 10^4^ cells/mL	2 cells/mL	[178]
DNAzyme	miR-155 and lysozyme	0.01 nM~1μM and 1 pM~1μM	5.2 pM and 0.67 pM	[179]
Fc-DNA	bleomycin	5~2000 pM	4 pM	[181]
Methylene blue-DNA	exosomes	10^3^~10^9^ particles/mL	708 particles/mL	[182]
DNA superstructure	exosomes	1 × 10^5^~3.7 × 10^8^ particles/mL	3.0 × 10^4^ particles/mL	[184]
HRP-MBs	CA125	2~100 U/mL	0.08 U/mL	[189]
Methylene blue-DNAmodified AuNPs	BNP	1~1 × 10^4^pg/mL	0.56 pg/mL	[194]
Fc-DNAmodified AuNPs	miRNA-182	0.001~2 pM	0.058 fM	[195]
Electroactive polymers	miRNA-21	1 aM~1 nM	0.32 aM	[196]

Abbreviations: LPs, liposomes; Fc, ferrocene; HIV-DNA, human immunodeficiency virus DNA; BChE, butyrylcholinesterase; HRP, horseradish peroxidase; OP-AChE, organophosphorylated acetylcholinesterase; PS, polystyrene; OTA, ochratoxin A; FB1, fumonisin B1; CEA, carcinoembryonic antigen; AFP, α-fetoprotein; *S. aureus*, *Staphylococcus aureus*; HAP, hydroxyapatite nanoparticle; ALP, alkaline phosphatase; Thi, thionine; CHA, catalytic hairpin assembly; CA, carbohydrate antigen; BNP, brain natriuretic peptide.

## 4. Conclusions

With the continuous development of nanotechnology, the preparation, functionalization, and application of magnetic materials have attracted extensive attention. In order to meet the growing demand for ultrasensitive detection of targets in complex samples, magnetically assisted electrochemical biosensors have been extensively developed in the fields of clinical diagnosis, food control, and environmental monitoring. MBs and MNPs as the magnetic cores to generate various function materials have become the important components of sensing transduction devices. To improve the sensitivity of magnetically assisted sensing protocols, different signal amplification strategies have been successfully integrated into bioassays, such as enzymes, DNA technology, and nanomaterials. In this work, we comprehensively summarized the design strategies of magnetically assisted electrochemical biosensors using magnetic materials as the platforms for recognition reaction and target conversion. We mainly focused on the representative examples of magnetically assisted electrochemical biosensors in terms of detection principle and analytical performances.

Despite the satisfactory achievements, there are still some challenges in the practical applications of magnetically assisted electrochemical biosensors. For example, in order to isolate and immobilize antibodies, enzymes, or other species, especially in the commercial field, it is necessary to prepare different batches of monodisperse magnetic materials and/or functional nanomaterials. In addition, the synthesis of magnetic nanostructured materials with more functions may open the door to develop novel magnetically assisted electrochemical methods with interesting transduction schemes. Moreover, most of the reported detection methods are carried out in the central laboratories, which should be optimized and integrated with microfluidic technology or disposable electrode arrays for applications in real life.

## Data Availability

Not applicable.
